# Towards Automated 3D Inspection of Water Leakages in Shield Tunnel Linings Using Mobile Laser Scanning Data

**DOI:** 10.3390/s20226669

**Published:** 2020-11-21

**Authors:** Hongwei Huang, Wen Cheng, Mingliang Zhou, Jiayao Chen, Shuai Zhao

**Affiliations:** Key Laboratory of Geotechnical and Underground Engineering, Department of Geotechnical Engineering, Tongji University, Siping Road 1239, Shanghai 200092, China; huanghw@tongji.edu.cn (H.H.); 1932289@tongji.edu.cn (W.C.); 1810181@tongji.edu.cn (J.C.); zhaos@tongji.edu.cn (S.Z.)

**Keywords:** water leakage, mobile laser scanning, point cloud, deep learning, 3D reconstruction, shield tunnel lining

## Abstract

On-site manual inspection of metro tunnel leakages has been faced with the problems of low efficiency and poor accuracy. An automated, high-precision, and robust water leakage inspection method is vital to improve the manual approach. Existing approaches cannot provide the leakage location due to the lack of spatial information. Therefore, an integrated deep learning method of water leakage inspection using tunnel lining point cloud data from mobile laser scanning is presented in this paper. It is composed of three parts as follows: (1) establishment of the water leakage dataset using the acquired point clouds of tunnel linings; (2) automated leakage detection via a mask-region-based convolutional neural network; and (3) visualization and quantitative evaluation of the water leakage in 3D space via a novel triangle mesh method. The testing result reveals that the proposed method achieves automated detection and evaluation of tunnel lining water leakages in 3D space, which provides the inspectors with an intuitive overall 3D view of the detected water leakages and the leakage information (area, location, lining segments, etc.).

## 1. Introduction

Metro shield tunnels have become important infrastructure in cities. As the tunnel service time increases and the environment inside the tunnel deteriorates, tunnel linings inevitably begin to suffer from various defects. Water leakage is one of the most common defects which requires regular inspection and evaluation for the purposes of tunnel health monitoring [1]. Several case studies [2,3] of severe tunnel damage caused by the delayed detection of water leakage have suggested that water leakage should be detected and located at an early stage. Meanwhile, remedial measures should be applied in time to prevent potential tunnel structural damage. Typically, only a short midnight period is spared for the tunnel lining defect inspection [4,5,6]. Thus, traditional manual inspection methods, marking the location and estimating the size of the water leakage, no longer meet the daily growing inspection requirements.

An automated, high-precision, and robust water leakage detection method is of great significance to improve the manual approach. Over the last two decades, two categories of non-destructive evaluation (NDE) approaches for tunnel leakage inspection have emerged: photogrammetry-based method using digital cameras to obtain two-dimensional (2D) images, and the light detection and ranging (LIDAR)-based method using acquired three-dimensional (3D) point cloud data [7]. These NDE methods overcome the inefficiency and high labor costs associated with the manual inspection method by efficiently extracting and processing water leakage features out of the tunnel lining surface data.

The photogrammetry-based methods process each tunnel lining image independently, and detect the corresponding water leakages on that specific 2D image. Therefore, it is challenging to visualize the detected water leakages in 3D space and obtain their spatial coordinates. In field practice, experienced inspectors usually locate the water leakage first and then evaluate the defects relative to the neighboring areas [8]. As an alternative to manual inspection, a 3D model of the tunnel linings provides a comprehensive visual perspective of detected water leakages. Although the structure from motion (SfM) technique can reconstruct a 3D model from 2D images, the 3D reconstruction performance depends greatly on the 2D image quality [9]. In an underground tunnel environment, the quality of the tunnel lining images is susceptible to the lighting conditions, camera position, and other environmental conditions [10]. Thus, an SfM-based 3D model cannot provide reliable spatial coordinates for water leakages, and the 2D image-based methods can only identify the responsible leakage defects in the images. To the authors’ knowledge, an automated method that can detect the water leakage and simultaneously obtain its 3D spatial coordinates is yet to be proposed.

In recent years, mobile laser scanning (MLS) has been one of the LIDAR technologies that has greatly improved the efficiency of inspections, and has gained popularity in tunnel inspection tasks [11,12]. MLS provides a 3D point cloud with high-precision spatial coordinates as well as the detailed features of the scanned tunnel lining surfaces. The water leakages are detected by converting the 3D point cloud data into 2D grayscale images. Then, the leakage features are further extracted by manual labeling or semi-automated image processing (IP). The manual labeling approach inevitably lacks objectivity due to naked-eye-detection during the time-consuming labeling process [13]. For the IP approach, various algorithms (e.g., Otsu algorithm [14], region growing algorithm [15], and edge detection [16], etc.) are used to detect structural defects more efficiently and reduce manual intervention. For water leakage detection, researchers [1,17] have applied the IP method to detect water leakage automatically using MLS technology. However, these above-mentioned IP methods have difficulty in achieving high detection precision due to existing interference features such as cables and bolt holes, which present similar grey image features as water leakage.

Towards automated water leakage detection, deep learning methods have begun to show great advantages in pixel level feature extraction [18]. In the last twenty years, convolutional neural networks (CNN) have emerged as an important branch of deep learning methods and have demonstrated strong capabilities in object detection tasks [19]. In civil engineering, the CNN-based method has been widely used in the detection of cracks [20], spalling [21], corrosion [22], deformation [23], and rock interlayers [24,25], etc., and has proven to have better precision and robustness than IP methods. Moreover, the development of popular and powerful CNN frameworks such as ResNet [26] and ResNext [27] has made it possible to extract the deeper semantic features of structural defects. For the automated water leakage detection, several CNN-based frameworks such as FCN [28], faster R-CNN [29], and R-FCN [30] have been proposed to achieve either semantic segmentation (classification at the pixel level) or object detection (classification and localization of the object) [31,32,33]. The results revealed that the CNN-based methods have great superiority over the IP. Instance segmentation, which combines semantic segmentation (i.e., DeepLabV3+ [34]) and object detection, has been considered a better approach for water leakage detection. Mask and region-based convolutional neural network (Mask R-CNN) [35] is easy to implement and is regarded as the most popular algorithm to realize instance segmentation, which has been used to achieve a high leakage detection efficiency and accuracy in the tunnel [36,37,38]. Path aggregation network (PANet) [39] has improved the feature information propagation path in Mask R-CNN, which is the state-of-the-art instance segmentation algorithm. Nevertheless, PANet has not demonstrated proven performance in detecting water leakages in shield tunnel linings. Therefore, Mask R-CNN was selected as the water leakage detection algorithm in this paper.

In order to visualize the spatial distribution information of the detected leakage, the detection results need to be projected into 3D space by reconstructing a surface model. Poisson reconstruction [40] was used by Ao et al. [41] and Liu et al. [42] to reconstruct the metro tunnel from a point cloud, but this method requires high density of the point cloud data. Triangulation is also a common method of 3D reconstruction by adding connections between points to form a triangle network. Nojima et al. [43] used the Delaunay triangulation method to reconstruct the surface of the tunnel, but the reconstruction result shows relatively low precision. Stent et al. [44] generated 3D mesh models from images to aid with the management, simulation, and visualization of tunnel defects in tunnel inspection projects, where the defect locations in 3D space can be conveniently extracted. Nevertheless, previous study [42] suggests that the existence of interference objects on the tunnel lining surface presents difficulty in reconstruction. There is still an urgent need to propose a unified algorithm, which can efficiently reconstruct a high precision 3D lining surface model.

**Figure 1:**
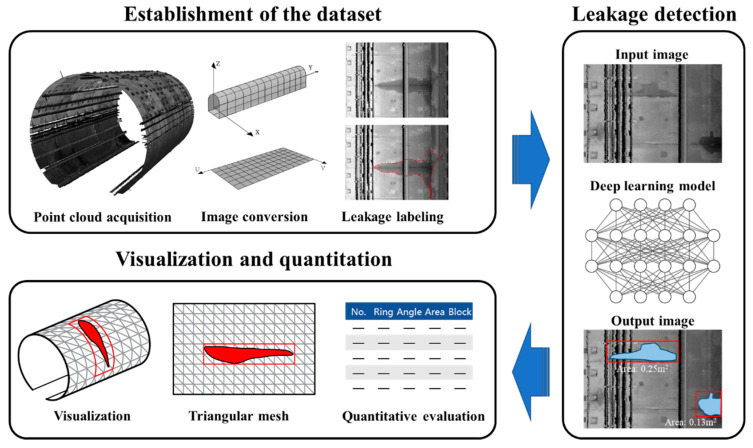
Schematic workflow of the proposed method.

In this study, an integrated method, combining a deep learning algorithm and MLS technology, is proposed to achieve automated 3D inspection of water leakages in shield tunnel linings. Figure 1 shows the workflow of the proposed method, which provides the inspectors with an intuitive overall 3D view of the detected water leakages and the leakage information. Overall, the major contributions of this study are as follows:

1. Establishment of the water leakage dataset, which entails point cloud data acquisition, grayscale image conversion, and ground truth water leakage labeling;
2. Automated water leakage detection using the Mask R-CNN algorithm, which demonstrates better accuracy and efficiency for the water leakage segmentation task than two state-of-the-art segmentation algorithms (PANet and DeepLabV3+);
3. 3D visualization and quantitation of the water leakages, where a novel triangular mesh method is proposed to efficiently generate a precise 3D tunnel lining model and automatically output a 3D inspection report containing the water leakage information and its spatial information.

The rest of the paper is organized as follows. The process of establishing the water leakage dataset is firstly presented in Section 2. Based on the dataset, automated water leakage detection and evaluation in 2D are presented in Section 3 and Section 4, respectively. A novel 3D reconstruction method is then proposed in Section 5, and 3D inspection results on testing samples are presented. To review the advancement and novelty of this study, three aspects of the proposed method were compared with the existing popular method in Section 6. Finally, the major findings are concluded in Section 7.

## 2. Water Leakage Dataset of Tunnel Linings

### 2.1. Point Cloud Data Acquisition Using MLS

**Figure 2:**
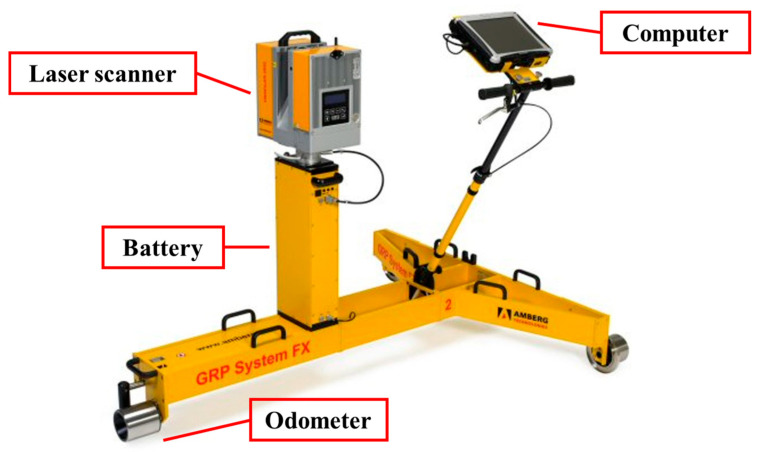
Schematic illustration of the mobile laser scanning (MLS) system (GRP5000).

**Figure 3:**
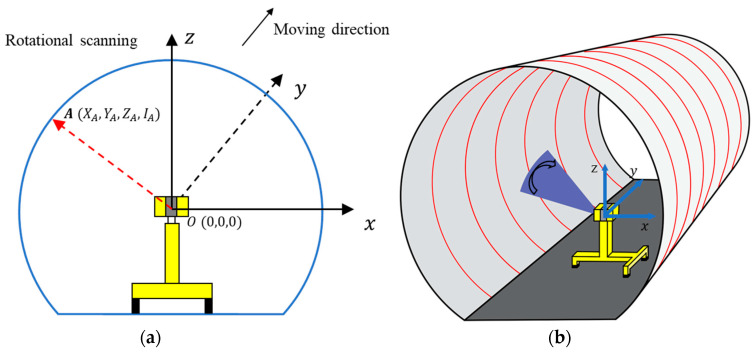
Relative coordinates of the MLS system: (**a**) principle of the tunnel profile scanning; (**b**) scanning in the 3D space.

The MLS system (GRP5000) produced by Amberg Technologies was used to collect tunnel lining point cloud data in this study. The GRP5000 system is integrated by a laser scanner, inspection vehicle, computer, battery, odometers, and other instruments. Of these, the inspection vehicle is the carrying platform of the entire system, which closely synchronizes various sensors with a scanner, as shown in Figure 2. Over 100 GB of point cloud data of a 4 km long Shanghai Metro tunnel were obtained by MLS, where each point contains the information of its coordinates and its intensity value (X, Y, Z, I). The coordinates are the relative position of the laser scanner (Figure 3), the Y axis represents the longitudinal direction towards which the GRP5000 system was moving, and the X axis and Z axis lie in the transverse section of the tunnel linings. The intensity value is positively correlated to the reflectivity of the object [45]:

(1) I∝ρ, 

where I denotes the intensity value recorded in 8 bits (0 to 255), and ρ is the reflectivity of the object on the tunnel lining.

### 2.2. Converting 3D Point Cloud into 2D Image

**Figure 4:**
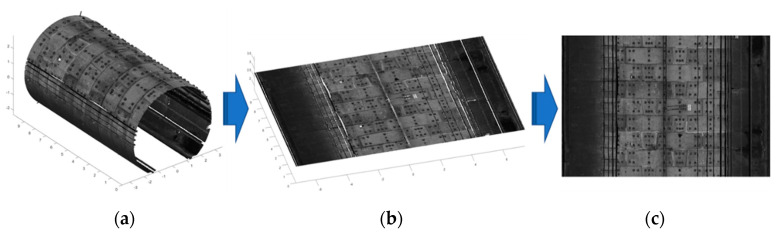
Conversion from 3D point cloud to 2D grayscale image, including: (**a**) 3D point cloud, (**b**) projected 2D point cloud, and (**c**) 2D grayscale image.

The acquired tunnel lining point cloud via the GRP5000 system contains a vast number of discrete and independent points. Hence, it is extremely difficult to detect and locate water leakages directly from the point cloud without any topological information. Using the assumed cylindrical shape of the metro shield tunnel, projection and gridding methods have been widely adopted to convert 3D point cloud data (X, Y, Z, I) into 2D grayscale images (U, V, GV) [1,13,46]. U and V indicate the location of each point in the 2D image matrix and GV is the corresponding grey value. The transformation method used in this work has two steps: (1) project the 3D point cloud onto a 2D surface, and (2) generate 2D grayscale tunnel lining images (Figure 4).

#### 2.2.1. 3D Point Cloud Unrolling

**Figure 5:**
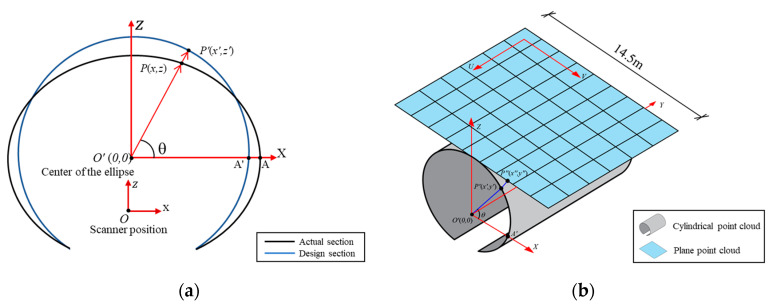
Point cloud unrolling: (**a**) cylindrical projection using the raw 3D point cloud data, and (**b**) converting the cylindrical point cloud onto the 2D plane.

The 3D point cloud is projected onto a 2D plane as shown in Figure 4b. The metro shield tunnel lining is generally designed as a standard cylindrical shape. After the inevitable small deformations of the tunnel, the transverse section of metro shield tunnel lining is usually assumed to be an ellipse [4]. The original coordinates system with respect to the laser scanner should be converted to the center of the ellipse of each cross-section (Figure 5a). The spatial coordinates of the point cloud can be corrected to the cylindrical shape (the design section of the tunnel lining) using perspective projection:

(2) $$\theta =∠POA=\cos^{-1}\left(\frac{\vec{OP}\cdot \vec{OA}}{\left|OP\right|\cdot \left|OA\right|}\right),$$

(3) $$x^{\prime }=R\cos\left(\theta \right),$$

(4) $$y^{\prime }=R\sin\left(\theta \right),$$

where R denotes the inner radius of the cylindrical tunnel (2.75 m), P(x,z) indicates a point of the raw point cloud, and $P^{\prime }(x^{\prime },z^{\prime })$ is the corresponding cylindrical point. Subsequently, the cylindrical-shape point cloud is unrolled along the Y axis to form a 2D point cloud according to the following equations:

(5) $$x^{\prime \prime }=\left(\frac{\pi }{2}-\theta \right)\times R,$$

(6) $$z^{\prime \prime }=R.$$

Through the unrolling process, the 2D point cloud with a width of 14.5 m is obtained (Figure 5b), and the coordinate of each point in the Y direction has not changed:

(7) $$y^{\prime \prime }=y^{\prime }=y.$$

#### 2.2.2. Generating 2D Grayscale Image

**Figure 6:**
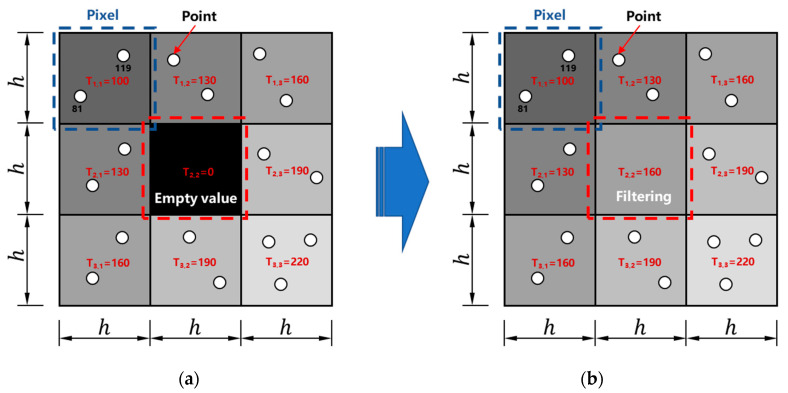
Calculation of grey value of the image: (**a**) before median filtering, and (**b**) after median filtering.

The grayscale image consists of m×n gray pixels with pixel values ranging from 0 (black) to 255 (white). To convert the 2D point cloud to the grayscale images, the 2D point cloud is first divided with a certain interval length (h) via square grid partition. The size of h controls the resolution of the output image. A smaller h results in a higher image resolution but may also create more empty value pixels where there are no points in the grid (Figure 6a). In this study, an appropriate size of h was assigned as 5 mm. Therefore, the output grayscale images with a resolution of 5 mm/pixel were generated. During this process, a 2D coordinate system (U and V) is generated with the top left pixel of the image as the origin as shown in Figure 5b. The U axis and V axis represent the longitudinal and transverse directions of the tunnel linings, respectively. The physical length of the laser scanned transverse section is 14.5 m, which corresponds to the width of the grayscale image and results in a resolution of 2900 pixels on the V axis. For all points in each 5 mm by 5 mm grid, the average intensity is used as the grey value of the corresponding grayscale image pixel. The grey value in each grid, $T_{u,v}$, is calculated as:

(8) $$T_{u,v}=\frac{\sum I_{u,v}}{n},$$

where n is the number of points in each grid, u and v indicate the location of point T in the grid. Moreover, a grid with an empty grey value is filled by a pixel value computed from the median filtering method [47], and the pixel value is defined as the median of the eight neighboring grey pixel values (Figure 6b):

(9) $$T_{u,v}=med\left(T_{u-1,v-1},T_{u-1,v},T_{u-1,v+1},T_{u,v-1},T_{u,v},T_{u,v+1},T_{u+1,v-1},T_{u+1,v},T_{u+1,v+1}\right).$$

### 2.3. Establishment of Tunnel Leakage Image Dataset

**Figure 7:**
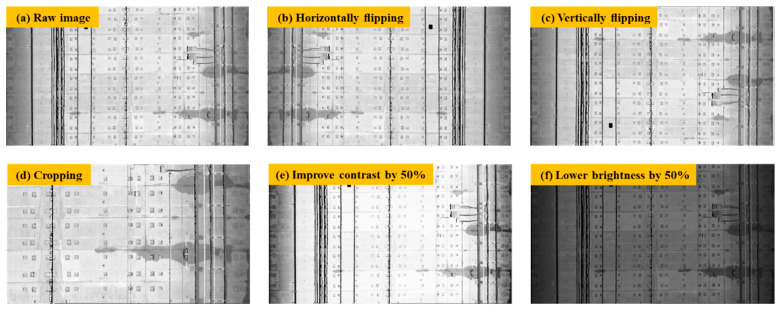
Data augmentation of the 2D grayscale image dataset.

**Figure 8:**
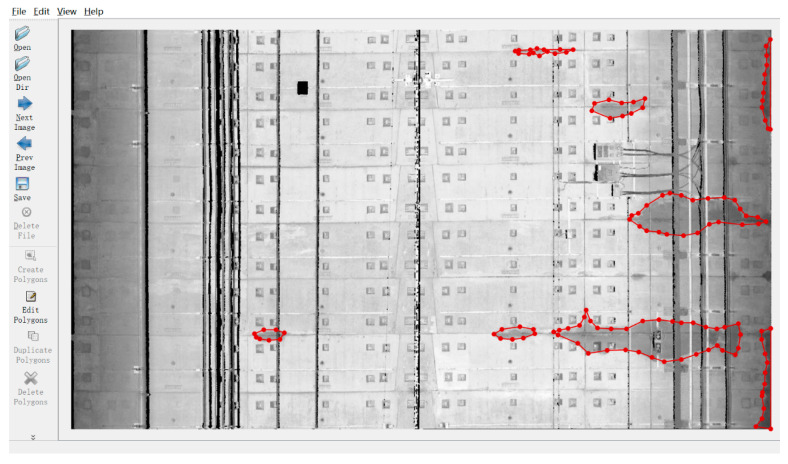
Water leakage annotated by LabelMe software.

2D grayscale images of the tunnel linings were generated based on the above-mentioned conversion method. After that, the 2D images were further selected and cropped into 275 images containing the water leakage by three sizes for training and validation (2900 × 2000 pixels, 2900 × 1600 pixels, and 2900 × 2400 pixels). Due to the limited number of water leakages in the inspected metro tunnel, it was challenging to obtain enough 2D images containing water leakage. According to Cui et al. [48], slight changes to the existing image dataset, such as flipping and rotating an image, can generate more images for training and validation purposes. Therefore, data augmentation was used to enlarge the water leakage image dataset (Figure 7). The 2D image dataset was enlarged from 275 images to 1650 images, and then divided into a training set, validation set, and testing set following a proportion of 7:2:1. Subsequently, all the water leakages in these 2D images were annotated by the open source software LabelMe [49], which is a graphical image annotation tool that labels polygons along the leakage boundaries (Figure 8). As required in the training process of the Mask R-CNN model, the annotated image data together with the 2D grayscale images were converted into the Microsoft COCO [50] datasets format to establish the required image dataset.

## 3. Automated Water Leakage Detection via Mask R-CNN

**Figure 9:**
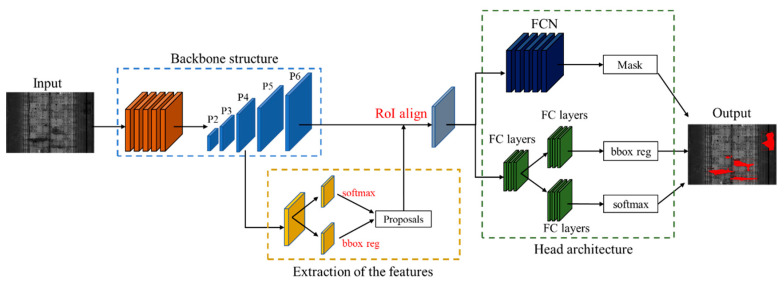
The overall structure of the mask and region-based convolutional neural network (Mask R-CNN) model.

Mask R-CNN, considered as the state-of-the-art of object detection algorithm, was developed on the basis of Faster R-CNN by adding region of interest (RoI) Align [35] and FCN [28]. The Mask R-CNN architecture is adopted in this study to realize the automated water leakage detection from the 2D images and then produce high-quality segmentation masks. Figure 9 illustrates the overall structure of the adopted Mask R-CNN, which includes the backbone architecture, region proposal network (RPN) [29], head architecture, and other components.

### 3.1. Backbone Structure

The employed Mask R-CNN model adopts ResNet-50 as the main backbone component, which contains 50 convolutional layers. A CNN model with a deeper network usually presents a greater capability of extracting the semantic features and a better prediction of performance [51]. In recent years, the CNN models using VGGNet [52], GoogleNet [53], and ResNet as the backbone have produced eye-catching performances on image detection tasks. Among those backbone structures, ResNet can effectively solve the problem of gradient explosion/vanishing in deep networks by establishing shortcut connections between convolutional layers.

A significant improvement in the backbone structure of the Mask R-CNN model is integrating the feature pyramid network (FPN) [54] and the convolutional layers to solve the problem of multi-scale object detection on images. FPN combines the advantages of the deeper layers and the higher resolution to build high-level semantic feature maps at all scales through a top-down architecture with lateral connections. The previous studies [31,32] using the other CNNs missed the opportunity to re-use the higher-resolution maps to extract the deeper semantic features when there is a fair amount of water leakage at different scales in the tunnel linings.

### 3.2. Extraction of the Features

Mask R-CNN uses the RPN to generate RoIs from the feature maps output by the backbone. The RPN uses the sliding windows to generate nine anchor boxes with different scales and aspect ratios in each pixel. These anchor boxes are further revised for a more accurate bounding-box via the regression branch. In typical two-stage detection frameworks such as Fast-RCNN and R-FCN, the RoI pooling is integrated with RPN to pool the RoIs in the feature map into a fixed-size result. However, this process may result in misalignments between RoIs and the extracted features. Therefore, the RoI Align is introduced to Mask R-CNN by replacing RoI pooling with bilinear interpolation, which solves the problem of misalignment caused by two quantization during RoI pooling.

### 3.3. Head Architecture

There are three parallel output branches in the head architecture of the Mask R-CNN network: the classification, bounding boxes regression, and mask branches. The classification branch outputs a vector $p=\left(p_{0},p_{1},\ldots ,p_{n}\right)$ representing the probability that a RoI belongs to each category or background. The bounding boxes regression branch outputs the coordinate of the bounding boxes by a vector $t=\left(t_{x},t_{y},t_{w},t_{h}\right)$ for each RoI. The mask branch uses an FCN to generate k masks for each predicted instance without competition between classes, and this approach is the key to improving the prediction results of the model.

### 3.4. Training and Testing

The Mask R-CNN code used in this study is an open source code in Detectron2, which is the latest version of the object detection platform developed by Facebook AI research [55]. The Microsoft COCO format dataset of water leakage in the metro tunnel was trained using a self-assembled desktop PC, which is configured with an Intel Core i7-9700k processor, 32GB RAM, Nvidia GTX 2080Ti 12GB GPU, and Ubuntu 18.04 operating system.

**Figure 10:**
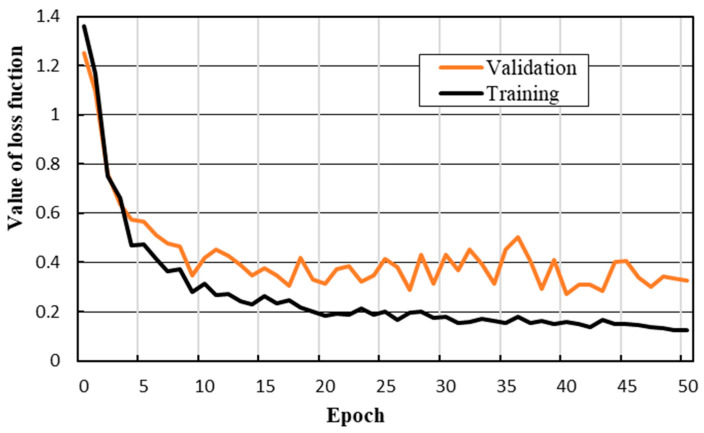
The loss value curve of the training and validation processes.

The initial settings of the hyperparameters (e.g., learning rate, number of epochs, etc.) have effects on the training stage. The learning rate refers to the step size at each iteration of updating the network weight. A high learning rate may lead the gradient descent to cross the optimal value and a low learning rate may take too long to converge or get stuck in an undesirable local minimum. The number of epochs represents the number of times that all the images are fed into the network to complete the network weight updating. The original number of epochs can be set large, and the optimal model can be determined and selected by the minimum loss value of the validation set in the training stage. In this paper, the initial learning rate of the training is 0.001, and the number of epochs is set to 50. After 20 epochs, the learning rate decays to 0.0001. As shown in Figure 10, the value of the loss function shows a downward trend throughout the training and validation process, which means that the prediction error decreases by updating the loss function with small batches. When the number of epochs is greater than 40, the loss values of the training set and the validation set gradually reach a state of convergence.

**Figure 11:**
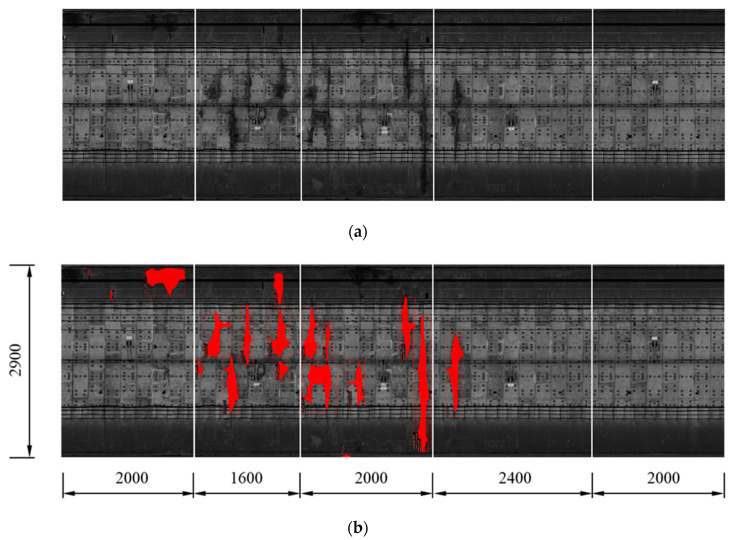
Results of the segmentation of tunnel leakage: (**a**) 5 testing tunnel lining images (a total resolution of 2900 × 10,000 pixels, i.e., a 50 m-long tunnel), and (**b**) testing results of the water leakage segmentation output from the Mask R-CNN model.

A total of 165 random selected leakage images in the established dataset were evaluated and tested. The testing results of consecutive images from the dataset can be stitched together to form a larger image that has multiple water leakages along a length of shield tunnel lining. Taking five images with a length of 50 m as an example (Figure 11a), most of the water leakages in the tunnel linings were detected and marked with the predicted segmentation masks.

## 4. Automated Water Leakage Evaluation

### 4.1. Water Leakage Evaluation in 2D

The water leakage area and its location are regarded as the two most important pieces of information during the evaluation of tunnel structural safety [56,57]. Therefore, the area of each water leakage and its spatial coordinates in the tunnel are computed based on the output images from the Mask R-CNN model containing water leakage information.

**Figure 12:**
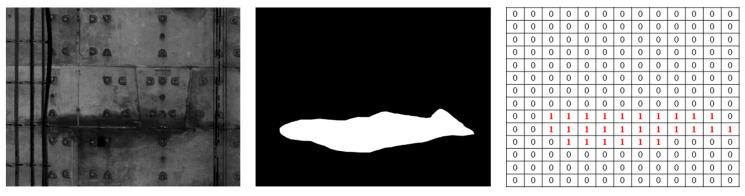
Illustration of the binary mask image.

The binary mask image for each instance is output by the mask branch through FCN, where the foreground (leakage mask) is assumed to be equal to 1 (white color in Figure 12), and the background (interference) is assumed to be equal to 0 (black color in Figure 12). Hence, the water leakage area is proportional to the number of white pixels and can be calculated as follows:

(10) $$A_{i}=n_{i}A_{p}=n_{i}h^{2},$$

where $A_{i}$ denotes the area of the water leakage, $n_{i}$ refers to the number of water leakage pixels, and $A_{p}$ represents the actual area for each pixel (25 mm^2^), which is the square of the grid interval (h, 5 mm) as introduced in Section 2.2.2.

### 4.2. Output Images from the Modified Mask R-CNN

**Figure 13:**
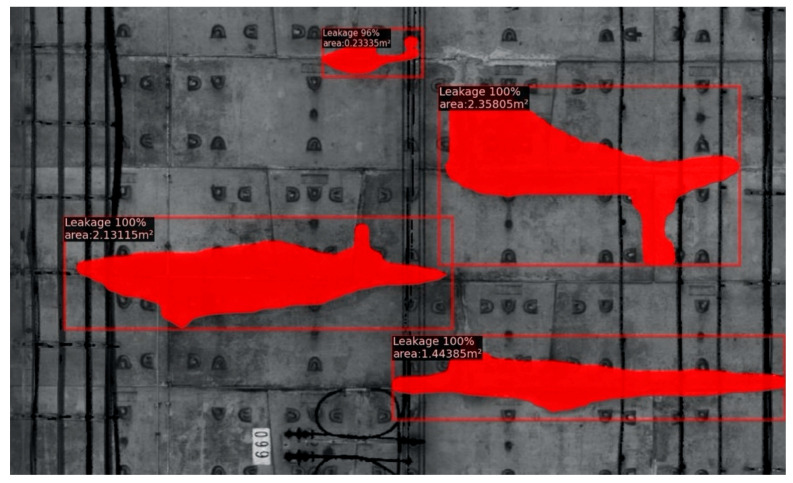
Water leakage evaluation in 2D.

The output from the original Mask R-CNN can only present the segmentation masks, confidence coefficient, and the bounding boxes. In order to present the water leakage evaluation results, it was modified to automate the 2D and 3D evaluation process. The 2D images output from the modified Mask R-CNN can show the following evaluation information: the segmentation masks, the leakage area, the confidence coefficient for object detection, and the predicted bounding boxes (Figure 13). As a result, the output data format from the modified Mask R-CNN code is (U, V, R, G, B), where U and V are the coordinates of each pixel point in the 2D image space. Meanwhile, the RGB pixel values are used to incorporate both the tunnel lining pixels and the pixels containing water leakage information (the masks, the evaluation results, the bounding box, etc.).

### 4.3. Water Leakage Evaluation in 3D

**Figure 14:**
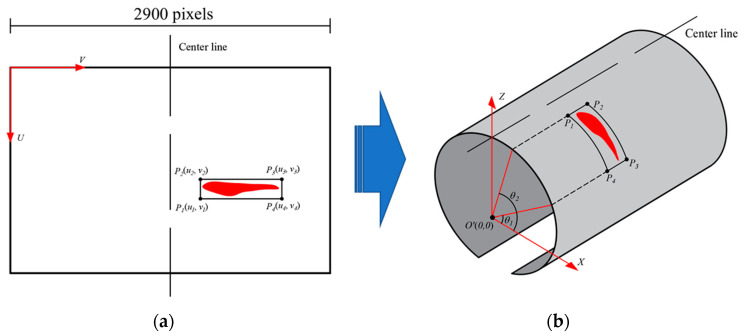
Obtaining the tunnel leakage location in 3D space: (**a**) water leakage positioning on the 2D image, and (**b**) transforming the 2D coordinates into 3D space.

The point cloud data has all the location information of the tunnel linings. Therefore, according to the projection relationship between the point cloud and the processed 2D images output from the Mask R-CNN model, the 3D coordinates of the water leakage pixels can be obtained from the 2D coordinates of each pixel. Figure 14 shows the process of the coordinate transformation. The center line of each 2D image is identified and considered to be the Z axis in the 3D space. Reversing the projection process in the unrolling step (Section 2), the 2D coordinates of each pixel are projected back onto the 3D cylindrical surface and the corresponding 3D coordinates are obtained.

**Figure 15:**
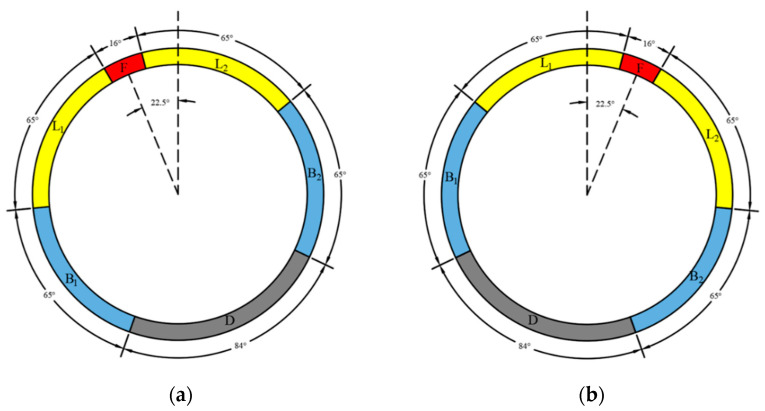
Staggered joint assembly of the shield tunnel in this study, where: (**a**) ring number is odd, and (**b**) ring number is even.

The 3D coordinates of the four vertices of a detected bounding box (P_1_, P_2_, P_3_, P_4_) are used to determine the location of each water leakage. The angles of the four vertices are obtained by converting the 3D Cartesian coordinates into 3D polar coordinates and using the center of the transverse section circle as the origin (Figure 14b). Based on the polar coordinates of the four vertices, the corresponding ring number of each water leakage can be derived from a prior known ring number in the system. As a case demonstration, the detected water leakage in a 50 m metro tunnel was evaluated (Figure 11). Table 1 summarizes the spatial distribution information of the water leakage, and the angles $\theta_{1}$ and $\theta_{2}$ (Figure 14b) can be calculated using the following equations:

(11) $$\theta =\frac{\pi }{2}-\frac{\left(v-1450\right)\times 0.05}{R},$$

(12) $$N=\left[\frac{u\times 0.05}{W}\right],$$

where the inner radius R and the ring width W of the shield tunnel lining are assumed to be 2.75 m and 1.2 m in the Shanghai metro tunnel, respectively, the values u and v denote the 2D coordinates of the vertices, and N is the relative ring number of a certain tunnel lining inspection region. The shield tunnel lining investigated in this study is generally composed of six segments: one capped block F, two adjacent blocks L_1_ and L_2_, two standard blocks B_1_ and B_2_, and one bottom block D [58,59] (Figure 15). In this case, the staggered joint assembly angles of lining ring are −22.5° for the odd numbered tunnel rings (Figure 15a) and 22.5° for the even numbered tunnel rings (Figure 15b). Thus, the calculated angle of the four vertices of a detected bounding box can be used to determine which segment block water leakage belongs to which ring, as shown in Table 1.

## 5. Visualizing the Water Leakage in 3D Space

**Figure 16:**
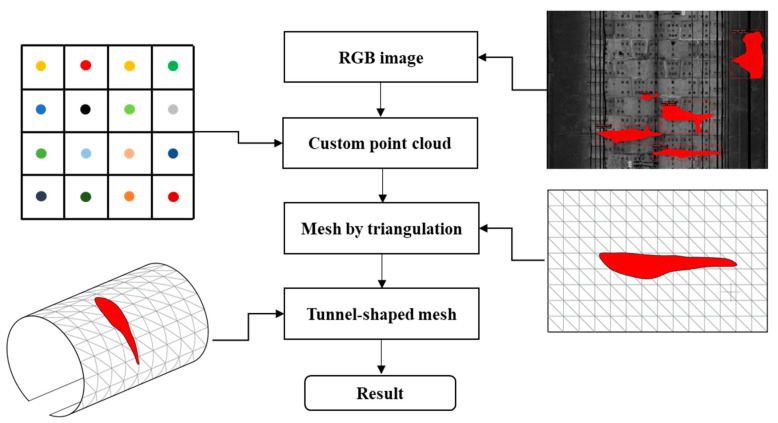
Workflow of the 3D tunnel surface reconstruction including water leakage information.

A novel 3D reconstruction method is proposed in this study, which can simultaneously fuse the point cloud and 2D images while imposing an explicit relationship between the discrete spatial points. The specific procedure of this reconstruction method contains three steps (Figure 16): (1) establish a custom 2D point cloud according to the RGB images; (2) adopt a triangular mesh method to generate 2D mesh from the 2D point cloud; and (3) perform the coordinates transformation from the 2D mesh to the 3D tunnel lining surface.

**Figure 17:**
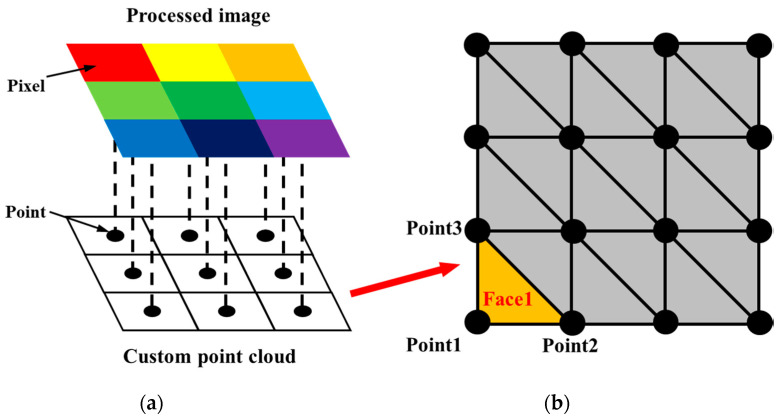
Custom 2D point cloud creation and generation of the 2D mesh: (**a**) generating the custom 2D point cloud from the RGB image, and (**b**) schematic diagram of the diagonal mesh method.

According to previous studies [42,43], the traditional 3D reconstruction methods have difficulties in performing a triangular mesh on the unordered spatial tunnel point cloud on account of the complex surface of the tunnel linings. Therefore, an effective solution is to create mesh in 2D space by projecting the 2D images onto the 2D plane point cloud. In this process, the output images from the modified Mask R-CNN containing water leakage information are used for generating the custom 2D RGB point cloud, and each pixel is represented as a point in the center of a grid (Figure 17). For consistency, each grid has a size of 5 mm in each direction. The custom 2D RGB point cloud can effectively reduce the number of points while retaining all the RGB information of the images. After that, the two nearest neighboring points of each point shall be connected, and a diagonal mesh method is adopted to create the 2D mesh.

The 2D plane mesh is then reconstructed as a 3D tunnel-shaped model. The 2D mesh is firstly rolled up and reconstructed as a cylindrical 3D surface model by reference to Equations (5)–(7). Then, the cylindrical 3D surface mesh is transformed into a 3D tunnel-shaped mesh and this procedure is achieved using the Algorithm 1 as shown below.
**Algorithm 1.** Converting the 2D mesh to a tunnel-shaped mesh**Input:** raw point cloud *R* (*x, y, z*); meshed plane point cloud *P* (*x, y,* 2.75)**Output:** tunnel-shaped mesh *T* (*x, y, z*)  1: *R* (*θ, y, ρ*) ← Switch Cartesian coordinates *R* (*x, y, z*) to polar coordinates  2: *P′* (*x′, y′, z′*) ← Roll up the plane data *P* (*x, y,* 2.75) to cylindrical data  3: *P″* (*θ″, y″, ρ″*) ← Switch Cartesian coordinates *P′* (*x′, y′, z′*) to polar  4: **for** each point *P″_i_* (*θ″_i_, y″_i_, ρ″_i_*) **do**  5:    Extract the set *N_k_* (*θ, y, ρ*) from *R* (*θ, y, ρ*) ← *k* nearest neighbors of *θ″_i_* where | *y″_i_ − y* | < *m*
  6:    Compute the average *ρ^k^_i_* from *N_k_* (*θ, y, ρ*)  7:    *T_i_* (*x_i_, y_i_, z_i_*) ← Switch polar coordinates (*θ″_i_, y″_i_, ρ^k^_i_*) to Cartesian coordinates  8: **end for**  9: **return**
*T*

**Figure 18:**
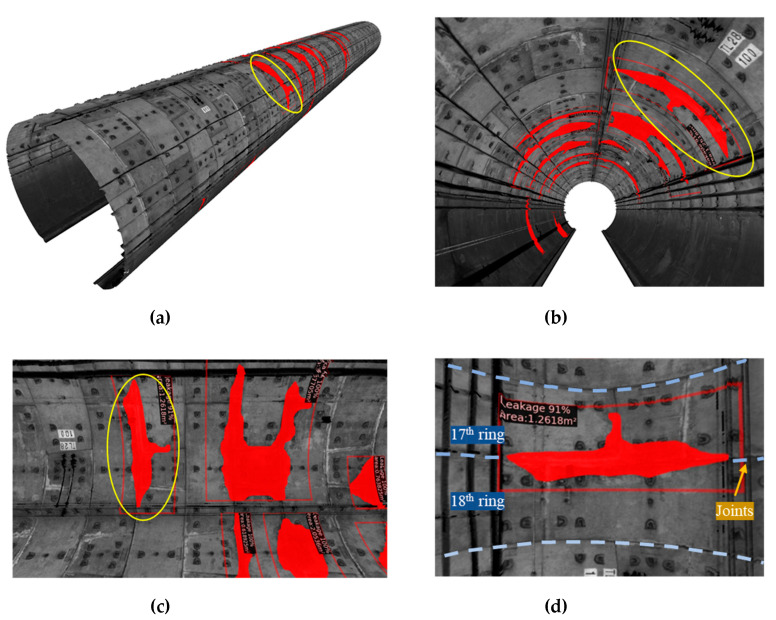
Surface reconstruction result of a 50 m shield tunnel lining model: (**a**) overall view of the 3D model, (**b**) longitudinal view, (**c**) front view of the detected water leakages, and (**d**) detailed water leakage information.

**Figure 19:**
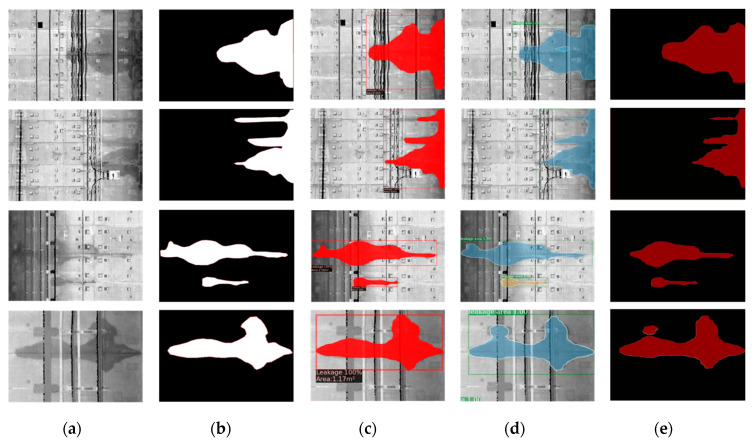
Four testing examples of segmentation results using three different methods. (**a**) Testing images; (**b**) ground truth; (**c**) Mask R-CNN; (**d**) PANet; (**e**) DeepLabV3+.

As a case study, a 50 meter shield tunnel lining model containing the detection information is generated. The 3D reconstruction result is shown in Figure 18. The same water leakage from a different viewing perspective is highlighted by the yellow circles in Figure 19a–c. The reconstructed 3D model of the tunnel can provide a first-person perspective view of the tunnel leakage, and intuitive observation of the location and distribution information of the water leakage. It can also be used for 3D roaming, which greatly improves the inspection quality by giving an overall view of the water leakages in shield tunnel linings.

## 6. Comparison and Discussion

Three aspects of the proposed method, namely water leakage segmentation, tunnel lining 3D reconstruction, and intuitive 3D inspection, were compared with the existing popular methods. The comparison results of the testing samples are presented and discussed to demonstrate the advancements and improved performances of the proposed method.

### 6.1. Segmentation Results Comparison

To further examine the segmentation performance of the trained Mask R-CNN model, the Mask R-CNN was compared with two state-of-the-art segmentation algorithms: PANet and DeepLabV3+. DeepLabV3+ is a pixel-to-pixel and end-to-end framework that can perform high precision semantic segmentation. The mean pixel accuracy (MPA), mean intersection over union (MIoU), and average inference time (AIT) are used in this paper to evaluate the performance of the different models:

(13) $$MPA=\frac{1}{w+1}\sum_{i=0}^{w}\frac{p_{ii}}{\sum_{j=0}^{w}p_{ij}},$$

(14) $$MIoU=\frac{1}{w+1}\sum_{i=0}^{w}\frac{p_{ii}}{\sum_{j=0}^{w}p_{ij}+\sum_{j=0}^{w}p_{ji}-p_{ii}},$$

where w+1 denotes the w classes and the background, and $p_{ij}$ represents the number of class i pixels which belong to class j. Similarly, $p_{ii}$ indicates the number of pixels that are correctly predicted. In other words, MPA refers to the ratio of correctly predicted pixels to all pixels, and MIoU represents the ratio of the interaction area to the union area between predicted pixels and the ground truth. Four testing images (Figure 19a) of the tunnel linings were selected and processed using three different segmentation algorithms (Mask R-CNN, PANet, and DeepLabV3+). The segmentation results of the three algorithms were compared against the labeled ground truth (Figure 19b), and the measured comparison metrics (MPA, MIoU, and AIT) are summarized in Table 2.

The DeepLabV3+ algorithm only performs semantic segmentation and outputs the classification of pixels. Hence, a binary water leakage segmentation map is produced (Figure 19e). However, instance segmentation algorithm (i.e., Mask R-CNN and PANet) can present both the leakage recognition results and the background information, which proves to be a better approach for leakage inspection. In terms of the metrics value, DeepLabV3+ has the lowest MPA and MIoU, and the longest AIT. Although PANet has more advanced neural network architecture than Mask R-CNN, the MPA and MIoU values of the water leakage segmentation obtained by Mask R-CNN and PANet are very close. Furthermore, in terms of computational efficiency, Mask R-CNN has the shortest AIT value (0.093 s/image) in comparison to PANet and DeepLabV3+. Overall, Mask R-CNN demonstrates high accuracy and efficiency for the water leakage segmentation task.

### 6.2. Reconstruction Results Comparison

In order to evaluate the reconstruction result, the proposed method was compared with two widely accepted 3D surface reconstruction algorithms: the Delaunay triangulation algorithm [43] and the Poisson reconstruction algorithm [40]. The Delaunay triangulation algorithm was realized by converting the 2D Delaunay triangulation mesh to a 3D space, and the Poisson reconstruction was implemented in the open source software MeshLab.

**Figure 20:**
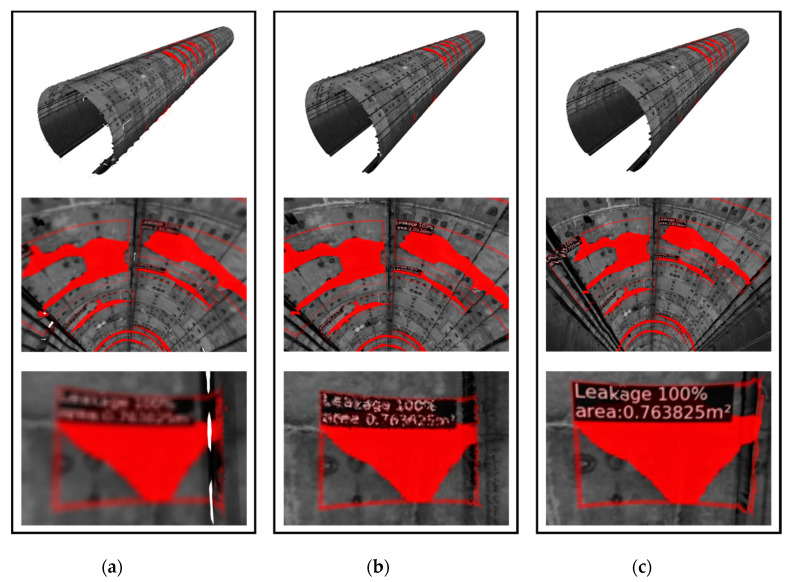
Results of tunnel reconstruction by different algorithms, including: (**a**) Poisson reconstruction, (**b**) Delaunay triangulation, and (**c**) the proposed method.

Figure 20 shows the result of a 50 m tunnel reconstruction between different algorithms, and the proposed method shows the best reconstruction performance in terms of resolution and precision. Poisson reconstruction is an algorithm that obtains the surface by solving the Poisson equation and then generating the iso-surface. The reconstructed model generates a relatively smooth surface and loses detailed features of the tunnel lining surfaces. Therefore, Poisson reconstruction is not suitable for high-precision tunnel point cloud reconstruction. The Delaunay triangulation algorithm generates a uniform and regular surface as well as the proposed method. However, it will lose the evaluation information of the water leakage printed on the 3D model because it lacks the custom 2D point cloud generation step.

Table 3 summarizes the key statics of the three reconstruction methods. Among them, the inference time of the Poisson reconstruction algorithm is the shortest, but it has also the least number of points and surfaces, which means that it cannot accurately restore the details of the tunnel lining surface. With the custom point cloud generation, the proposed method greatly reduces the number of reconstructed points on the premise of retaining all the RGB information. As a result, it has advantages in both speed and resolution compared with Delaunay triangulation. Overall, the proposed reconstruction method can generate a precise 3D tunnel lining model with decent efficiency.

### 6.3. 2D and 3D Inspection Results Comparison

**Figure 21:**
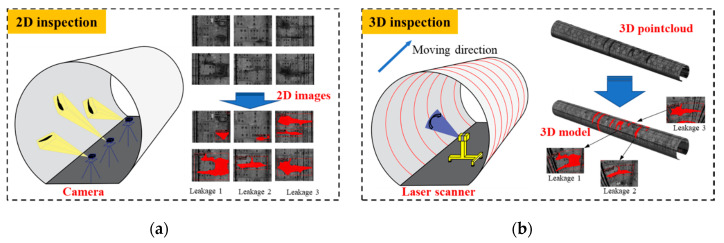
Differences of tunnel leakage inspection and visualization between: (**a**) a typical 2D inspection method, and (**b**) the proposed 3D inspection method.

A typical 2D inspection method detects water leakage in the tunnel linings by firstly taking 2D photographs of the lining surface (Figure 21a). After that, the obtained discrete and independent 2D images containing the water leakages are evaluated and outputted to the inspection report. As a result, the 2D inspection results lack intuitive perception of the detected water leakage, which ignores the 3D spatial coordinates of the leakages and their relative position information. However, the proposed 3D inspection method retains all the RGB information of pixels (i.e., water leakage evaluation information) and the 3D spatial geometric characteristics of the tunnel lining point cloud. Therefore, the target lining segment block and 3D coordinates of each water leakage can be detected simultaneously (Figure 21b) by fusing the processed images and the 3D point cloud. In this regard, the proposed method provides an intuitive overall view of the detected water leakages in a 3D tunnel lining model. Moreover, the water leakage information and its spatial location can be automatically outputted to an inspection report together with the intuitive 3D model.

## 7. Conclusions

Due to the limitations of 2D images, the traditional photogrammetry-based method generally lacks analysis for the three-dimensional positioning of defects in tunnel linings. For this purpose, the main contribution of this paper is the proposal of a novel 3D water leakage detection and evaluation method, which can automatically detect, quantify, and visualize water leakage in tunnel linings in 3D space. The proposed method integrates the MLS technology with a modified Mask R-CNN and a triangulation-based 3D reconstruction algorithm, and provides tunnel inspectors with an intuitive overall 3D view of the detected water leakages and the leakage information.

The major findings of this study can be concluded as following:

1. A water leakage dataset was established by collecting tunnel lining point cloud data from a 4 km metro tunnel section in Shanghai, China. The coordinates transformation and square grid partition approaches were used to achieve 2D image conversion, and data augmentation was adopted to help improve the performance of the trained model.
2. Based on the dataset, the Mask R-CNN algorithm was adopted to achieve automated evaluation of the water leakage (the masks, the evaluation results, the bounding box, etc.). In comparison with two state-of-the-art segmentation algorithms (PANet and DeepLabV3+), Mask R-CNN demonstrates better accuracy and efficiency for the water leakage segmentation task.
3. A novel triangular mesh method is proposed in this study to generate a precise 3D tunnel lining model with decent efficiency. The reconstruction result demonstrates sound performance for 3D visualization of the detected leakage, which retains the spatial geometric characteristics of the tunnel lining point cloud and the printed water leakage evaluation information.
4. The proposed 3D inspection method provides an overall view of the detected water leakages. The water leakage information and its spatial location information (the ring number, the leakage area, the angle scope, and the lining segments) can be automatically outputted to an inspection report together with the 3D tunnel lining model.

In future work, the precision of leakage detection needs to be further improved. The resolutions of the point cloud generated 2D images were 5 mm/pixel in this study, which cannot capture detailed defect information in the tunnel linings. In the authors’ previous research [31,38], six high-resolution linear charge coupled device (CCD) cameras were used to obtain 0.29 mm/pixel resolution images of the tunnel lining, which are available for high resolution leakage detection. Future research will focus on combining the CCD cameras and MLS technology for high-precision defects detection and evaluation in 3D space.

## Figures and Tables

**Table 1:** The evaluation result of the water leakages in a 50 m shield tunnel lining.

Leakage	Ring Number *N*	$\mathrm{Angle}\;\theta_{1}$$\sim \theta_{2}$ (°)	Area (m^2^)	Lining Segments
#1	3	−50~−45	0.06	D
#2	4	−19~−8	0.05	B_2_
#3	6~9	−54~−13	3.43	B_2_~D
#4	9~10	91~112	0.23	L_1_~L_2_
#5	10~11	15~83	2.36	L_1_~B_2_
#6	11~12	84~170	2.13	B_1_~L_2_
#7	12~13	4~96	1.44	L_1_~B_2_
#8	14~15	12~83	2.15	L_1_~B_2_
#9	14~15	−47~2	1.16	L_2_~D
#10	14~15	92~120	0.76	F~L_2_
#11	16~17	8~86	2.05	L_1_~B_2_
#12	16~18	98~165	3.38	B_1_~L_2_
#13	17~18	31~90	0.62	L_1_~B_2_
#14	18~19	237~240	0.06	D~B_1_
#15	19~20	94~159	1.26	B_1_~L_2_
#16	22~23	−9~86	1.86	L_1_~B_2_
#17	23~24	19~228	4.51	D~B_2_
#18	23~24	162~233	1.33	D~L_1_
#19	25~26	51~170	2.90	B_1_~L_2_

**Table 2:** Metrics of evaluation results with different algorithms (mean pixel accuracy (MPA), mean intersection over union (MIoU), and average inference time (AIT)).

Method	MPA (%)	MIoU (%)	AIT (s/Image)
Mask R-CNN	97.18	77.05	0.093
PANet	97.57	77.34	0.112
DeepLabV3+	95.34	75.84	0.486

**Table 3:** Comparison of 50 m tunnel reconstructions by different algorithms.

Algorithm	Raw Points	Reconstructed Points	Inference Time
Delaunay triangulation	13,623,722	13,623,722	560 s
Poisson reconstruction	13,623,722	1,669,747	121 s
Proposed method	13,623,722	7,250,000	334 s
